# Cardiovascular magnetic resonance imaging feature tracking: Impact of training on observer performance and reproducibility

**DOI:** 10.1371/journal.pone.0210127

**Published:** 2019-01-25

**Authors:** Sören J. Backhaus, Georg Metschies, Marcus Billing, Johannes T. Kowallick, Roman J. Gertz, Tomas Lapinskas, Burkert Pieske, Joachim Lotz, Boris Bigalke, Shelby Kutty, Gerd Hasenfuß, Philipp Beerbaum, Sebastian Kelle, Andreas Schuster

**Affiliations:** 1 University Medical Center Göttingen, Department of Cardiology and Pneumology, Georg-August University, Göttingen, Germany; 2 German Center for Cardiovascular Research (DZHK), partner site Göttingen, Göttingen, Germany; 3 University Medical Center Göttingen, Institute for Diagnostic and Interventional Radiology, Georg-August University, Göttingen, Germany; 4 German Heart Center Berlin (DHZB), University of Berlin, Department of Internal Medicine / Cardiology, Charité Campus Virchow Clinic, Berlin, Germany; 5 DZHK (German Centre for Cardiovascular Research), Partner Site Berlin, Germany; 6 Charité Campus Benjamin Franklin, University Medical Center Berlin, Department of Cardiology and Pneumology, Berlin, Germany; 7 Children's Hospital and Medical Center, University of Nebraska College of Medicine, Omaha, United States of America; 8 Hanover Medical School, Department of Pediatric Cardiology and Intensive Care, Hanover, Germany; 9 Department of Cardiology, Royal North Shore Hospital, The Kolling Institute, Nothern Clinical School, University of Sydney, Sydney, Australia; Faculty of Medical Science - State University of Campinas, BRAZIL

## Abstract

**Background:**

Cardiovascular magnetic resonance feature tracking (CMR-FT) is increasingly used for myocardial deformation assessment including ventricular strain, showing prognostic value beyond established risk markers if used in experienced centres. Little is known about the impact of appropriate training on CMR-FT performance. Consequently, this study aimed to evaluate the impact of training on observer variance using different commercially available CMR-FT software.

**Methods:**

Intra- and inter-observer reproducibility was assessed prior to and after dedicated one-hour observer training. Employed FT software included 3 different commercially available platforms (TomTec, Medis, Circle). Left (LV) and right (RV) ventricular global longitudinal as well as LV circumferential and radial strains (GLS, GCS and GRS) were studied in 12 heart failure patients and 12 healthy volunteers.

**Results:**

Training improved intra- and inter-observer reproducibility. GCS and LV GLS showed the highest reproducibility before (ICC >0.86 and >0.81) and after training (ICC >0.91 and >0.92). RV GLS and GRS were more susceptible to tracking inaccuracies and reproducibility was lower. Inter-observer reproducibility was lower than intra-observer reproducibility prior to training with more pronounced improvements after training. Before training, LV strain reproducibility was lower in healthy volunteers as compared to patients with no differences after training. Whilst LV strain reproducibility was sufficient within individual software solutions inter-software comparisons revealed considerable software related variance.

**Conclusion:**

Observer experience is an important source of variance in CMR-FT derived strain assessment. Dedicated observer training significantly improves reproducibility with most profound benefits in states of high myocardial contractility and potential to facilitate widespread clinical implementation due to optimized robustness and diagnostic performance.

## Introduction

CMR represents the reference standard in the assessment of cardiac morphology and function [1] without the limit of anatomical plane restrictions [2, 3]. Introduced in 2009, cardiovascular magnetic resonance feature tracking (CMR-FT) allows quantification of myocardial deformation on routinely acquired b-SSFP cine images [3, 4]. CMR-FT is extensively used in cardiovascular research and increasingly in clinical practice. It allows for comprehensive and reliable assessments of cardiac function [5–10] and has been applied to a broad range of cardiovascular diseases such as patients with dilated [11, 12] or ischemic [13] cardiomyopathy, after myocardial infarction [14–16] and in patients with complex cardiac malformations such as Ebstein’s Anomaly [17]. Raising evidence points towards incremental additional value of myocardial deformation assessment for clinical decision making beyond established markers for cardiovascular risk including left ventricular ejection fraction (LVEF) [10–12, 14, 18, 19]. However, the results of clinical trials employing CMR-FT usually arise from highly trained research unit core-laboratories and reproducibility amongst other centres employing CMR imaging in their clinical routine may differ. Studies involving CMR derived volumes and mass identify intra- and inter-observer variability as a leading source of bias [20–22] with efficient training being the major determinant to overcome this limitation [22]. Whilst recent data demonstrates the necessity of experience using CMR-FT for reliable strain assessment [23], data on the value of sufficient training is lacking. Consequently, we aimed to determine the impact and benefits of training on the reproducibility and variability of CMR-FT employing different commercially available software solutions.

## Methods

### Study population

The study population consisted of 12 heart failure (HF) patients including heart failure with preserved (HFpEF, n = 7) and reduced (HFrEF, n = 5) ejection fraction as well as 12 healthy volunteers. All patients gave written informed consent. The study was approved by the Ethics Committee of the Charité-University Medicine Berlin and complied with the Declaration of Helsinki. All individuals gave written informed consent before participating in the study.

### Cardiovascular magnetic resonance imaging

The CMR imaging protocol was employed on a clinical Philips Achieva 1.5 Tesla MR scanner. Electrocardiogram (ECG)-gated b-SSFP cine sequences were acquired for long-axis 2- and 4- chamber views (CV) as well as a short axis stack. Imaging parameters were as follows: 40 frames/cardiac cycle, pixel spacing 0.8mm x 0.8mm, 8mm slice thickness as well as inter-slice gap, TE 1.5ms, TR 3ms. LVEF was assessed in the SA stack [1].

### Feature-tracking

**Fig 1:**
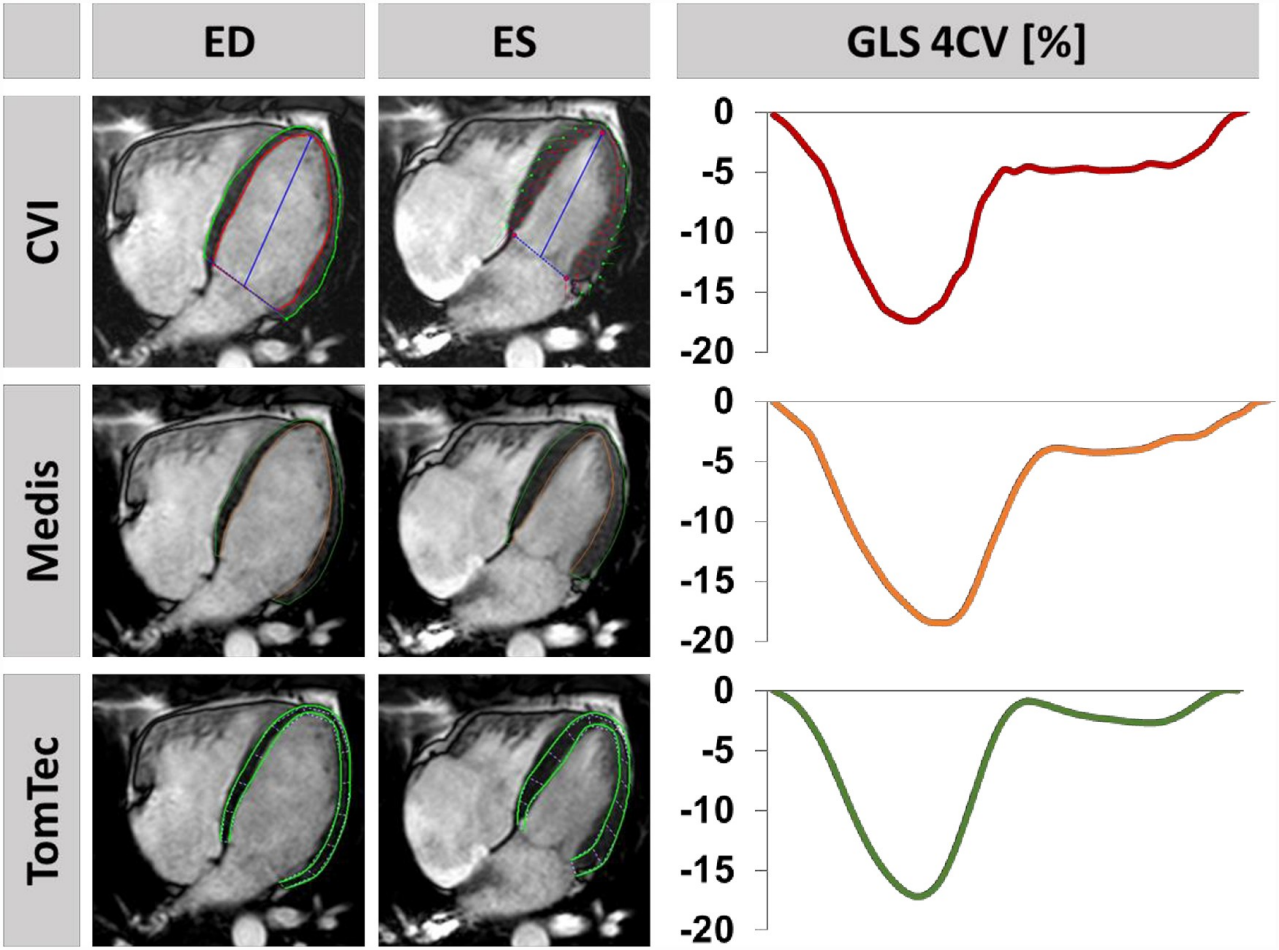
Feature-tracking using different software solutions. On the left, endo- and epicardially tracked borders of the left ventricle in a 4 chamber view (CV) at the end-diastole (ED) and end-systole (ES) are shown in a healthy volunteer using the different commercially available software solutions (upper row: CVI, middle row: Medis, bottom row: TomTec). On the right, the corresponding global longitudinal strain (GLS) curves are displayed.

CMR-FT based strain analyses were performed using commercially available software provided by 1. “CVI” (cvi^42^, Version 5.6.5., Circle Cardiovascular Imaging Inc., Calgary, Canada), 2. “Medis” (QStrain, Version 2.1.12.2, Medis Medical Imaging Systems, Leiden, Netherlands) and 3. “TomTec” (2D CPA MR, Version 4.6.3.9, TomTec GmbH, Unterschleissheim, Germany) (Fig 1). FT was performed in the end-diastole, and additionally in the end-systole using Medis. The LV was tracked at the endo- and epicardial borders. RV borders were tracked similarly using CVI, however in Medis and TomTec only an endocardial contour was applied. The tracking algorithms were then applied tracking tissue features over the cardiac cycle. Tracking accuracy was visually reviewed and if needed corrections were made to the initial contours only. This procedure was repeated for 3 times with subsequent averaging [8, 9]. Assessment included LV and RV global longitudinal strain (GLS), as well as global circumferential and radial (GLS/GRS) strain of the LV. The LV was tracked in 2- and 4- long axis CV with subsequent averaging of peak strain values to derive GLS. In opposite RV strain was derived from the 4-CV only [8]. Global short axis (SA) strain values (GCS and GRS) were averaged from 3 different slices identified at the basal (last slice with complete circular myocardium in absence of the left ventricular outflow tract), midventricular (level of both papillary muscles) and apical level (maintained blood-pool throughout the entire cardiac cycle).

### Work schedule

Strain analyses were performed by 2 inexperienced operators in patients and healthy volunteers.

Both operators had been exposed to some CMR imaging but not in active reporting or scanning and had no experience in deformation imaging. One operator focused more on basic the other on clinical science, both with an experience of 3 to 4 years in their field. Before the first tracking cycle, they have therefore been introduced to the different software solutions tested as well as how to technically correct start and apply these software solutions to which cine images. They have not been introduced to details in neither performing nor interpreting deformation imaging. To assess observer depending variability, all cases were analysed twice before and twice after dedicated observer training with at least 4 weeks in between each analysis to avoid recollection effects [22]. The operators underwent a training of 1 hour delivered by a trained investigator at the core-laboratory immediately after the second tracking cycle, that is 1 month prior to cycle 3 to avoid impact on short-term memory effects. Training comprised the introduction to different types of strain including long axis as well as short axis strains. It further included an explanation of resulting strain curves, their different compositions regarding systolic and diastolic function. Additionally, operators have been introduced to software specific and anatomical difficulties and their impact on absolute strain values. Intra-observer and inter-observer reproducibility were calculated within and between the 2 inexperienced operators prior to and after training. To generate a reference value for inter-vendor comparison, control tracking was performed by the trained investigator at the core-laboratory, with proven excellent intra- and inter-observer reproducibility in previous trials [5–8, 24]. All operators were blinded to each other’s results.

### Statistics

Statistical analyses were performed using IBM SPSS Statistic Software Version 24 for Windows (IBM, Armonk, NY, USA) and Microsoft Excel. Continuous parameters are presented as mean ± standard deviation (SD). Intra- and inter-observer variability were assessed using Bland-Altman analyses [mean difference between measurements with 95% confidence interval (CI)] [25], intra-class correlation coefficients (ICC) based on a model of absolute agreement, considered excellent if ICC >0.74, good between 0.60 and 0.74, fair between 0.4 and 0.59 and poor below 0.4 as well as the coefficient of variation (CoV, SD of mean difference divided by the mean $\frac{SD(MD)}{mean}$) [7]. Dependent continuous parameters were tested using the Wilcoxon signed-rank test after testing for normal distribution using the Shapiro-Wilk test. Reliable identification of impaired LVEF below 50% by the means of LV GLS and GCS was evaluated using AUC analysis. P-values provided are two-sided, an alpha level of 0.05 and below was considered statistically significant.

## Results

### Demographics

12 HF patients consisting of 7 HFpEF (EF median 59, SD 5.7) and 5 HFrEF (EF median 37.0, SD 7.0) patients and 12 volunteers were enrolled (Table 1). There were no significant differences in gender between healthy volunteers and HF patients. The latter were significantly older (p<0.001) with decreased LVEF (p = 0.039) and strain values compared to healthy volunteers (S1 Table). Training resulted in a significant change in absolute strain values obtained for LV GLS Medis and TomTec and GCS using TomTec only.

**Table 1:** Patients demographics and characteristics.

Gender (F/M)		Vol.: 6/6 –Pat.:5/7		p
Age in years		Vol.:29 (5.0)–Pat.: 74 (6.5)		
LVEF in %		Vol.:60 (1.3)–Pat.: 50 (12.7)		
	Software	Before Training	After Training	
LV GLS (%)	CVI	-17.5 (5.53)	-16.2 (2.74)	0.224
	Medis	-18.4 (3.61)	-19.9 (3.58)	**<0.001**
	TomTec	-20.1 (4.11)	-19.1 (3.57)	**0.02**
GCS (%)	CVI	-18.4 (3.65)	-18.4 (3.97)	0.495
	Medis	-28.0 (7.08)	-28.4 (6.71)	0.226
	TomTec	-25.3 (5.90)	-25.7 (5.91)	**0.037**
GRS (%)	CVI	35.9 (8.86)	36.5 (9.18)	0.064
	Medis	50.3 (15.3)	50.7 (15.7)	0.935
	TomTec	25.3 (9.07)	23.9 (6.37)	0.361
RV GLS (%)	CVI	-22.9 (4.36)	-23.0 (3.78	0.992
	Medis	-23.6 (4.58)	-24.3 (5.14)	0.434
	TomTec	-26.6 (5.01)	-26.9 (5.34)	0.525

Continuous variables are expressed as mean (standard deviation). The Wilcoxon signed-rank test was used to determine significant differences for continuous and the chi-squared test for categorial variables. LVEF/RVEF, left/right ventricular ejection fraction; GLS/GCS/GRS, global longitudinal/circumferential/radial strain.

### CMR-FT reproducibility

Mean differences as well as corresponding SD, ICC and CoV of assessed strain values are provided in detail for CVI (Table 2), Medis (Table 3) and TomTec (Table 4). Bland-Altman plots are presented in the Supporting Information (S1 to S6 Figs).

**Table 2:** Intra- and inter-observer reproducibility using CVI prior to and after training.

Software: CVI	Strain	Mean Difference (SD of the Diff.)	ICC (95% CI)	CoV (%)
Intra-observer	LV GLS %	0.03 (0.87)	0.98 (0.95–0.99)	5.4
before Training	GCS %	-0.07 (0.75)	0.99 (0.98–1)	4.2
	GRS %	0.46 (2.03)	1 (0.99–1)	5.8
	RV GLS %	0.23 (2.50)	0.89 (0.77–0.95)	10.5
Intra-observer	LV GLS %	0.18 (0.60)	0.99 (0.97–0.99)	3.8
after Training	GCS %	0.24 (0.53)	0.99 (0.98–1)	2.9
	GRS %	-0.36 (1.30)	1 (1)	3.7
	RV GLS %	0.17 (1.86)	0.95 (0.90–0.98)	8.0
Inter-observer	LV GLS %	-0.4 (2.56)	0.86 (0.69–0.94)	16.2
before Training	GCS %	0.57 (1.05)	0.97 (0.94–0.99)	5.7
	GRS %	-0.85 (2.25)	1 (0.99–1)	6.3
	RV GLS %	-1.68 (5.14)	0.58 (0.12–0.81)	22.4
Inter-observer	LV GLS %	0.6 (1.06)	0.96 (0.90–0.98)	6.6
after Training	GCS %	1.08 (0.82)	0.97 (0.86–0.99)	4.4
	GRS %	-3.18 (2.06)	0.99 (0.77–1)	5.6
	RV GLS %	-0.21 (3.12)	0.86 (0.68–0.93)	13.6

SD: standard deviation. ICC: intraclass correlation coefficient. CoV: coefficient of variation. LV: left ventricular. RV: right ventricular. GLS: global longitudinal strain. GCS: global circumferential strain. GRS: global radial strain.

**Table 3:** Intra- and inter-observer reproducibility using Medis prior to and after training.

Software: Medis	Strain	Mean Difference (SD of the Diff.)	ICC (95% CI)	CoV (%)
Intra-observer	LV GLS %	-0.04 (1.91)	0.94 (0.86–0.97)	10.2
before Training	GCS %	-0.97 (3.31)	0.93 (0.83–0.97)	12.7
	GRS %	-0.82 (10.9)	0.86 (0.68–0.94)	23.0
	RV GLS %	0.37 (2.59)	0.89 (0.75–0.95)	11.7
Intra-observer	LV GLS %	-0.09 (1.36)	0.96 (0.91–0.98)	6.8
after Training	GCS %	-1.22 (1.43)	0.98 (0.86–0.99)	5.4
	GRS %	2.11 (8.0)	0.84 (0.63–0.93)	18.7
	RV GLS %	-0.07 (3.22)	0.76 (0.44–0.90)	13.9
Inter-observer	LV GLS %	-0.72 (2.92)	0.81 (0.56–0.92)	15.8
before Training	GCS %	2.95 (4.26)	0.86 (0.54–0.95)	15.2
	GRS %	-6.49 (13.25)	0.73 (0.37–0.88)	26.3
	RV GLS %	3.06 (3.54)	0.70 (0.08–0.89)	15.0
Inter-observer	LV GLS %	0.04 (1.61)	0.95 (0.88–0.98)	8.1
after Training	GCS %	2.85 (2.48)	0.92 (0.33–0.98)	8.7
	GRS %	-13.97 (11.25)	0.64 (0–0.88)	22.2
	RV GLS %	3.33 (4.72)	0.58 (0.02–0.82)	19.1

SD: standard deviation. ICC: intraclass correlation coefficient. CoV: coefficient of variation. LV: left ventricular. RV: right ventricular. GLS: global longitudinal strain. GCS: global circumferential strain. GRS: global radial strain.

**Table 4:** Intra- and inter-observer reproducibility using TomTec prior to and after training.

Software: TomTec	Strain	Mean Difference (SD of the Diff.)	ICC (95% CI)	CoV (%)
Intra-observer	LV GLS %	-1.24 (1.52)	0.95 (0.87–0.98)	7.6
before Training	GCS %	-0.53 (1.3)	0.99 (0.97–1)	5.1
	GRS %	-0.70 (2.92)	0.99 (0.98–1)	12.1
	RV GLS %	-0.03 (2.93)	0.94 (0.87–0.97)	11.3
Intra-observer	LV GLS %	0.00 (1.07)	0.98 (0.95–0.99)	5.7
after Training	GCS %	0.35 (0.81)	1 (0.99–1)	3.1
	GRS %	1.42 (2.47)	0.99 (0.98–1)	10.2
	RV GLS %	0.08 (1.95)	0.98 (0.95–0.99)	7.3
Inter-observer	LV GLS %	-1.0 (2.43)	0.90 (0.77–0.96)	12.1
before Training	GCS %	-0.61 (1.60)	0.98 (0.96–0.99)	6.3
	GRS %	-3.22 (7.59)	0.94 (0.87–0.98)	30.0
	RV GLS %	1.35 (2.78)	0.93 (0.84–0.97)	10.5
Inter-observer	LV GLS %	1.03 (2.0)	0.91 (0.79–0.96)	10.5
after Training	GCS %	0.40 (1.37)	0.99 (0.97–0.99)	5.3
	GRS %	1.87 (3.53)	0.98 (0.96–0.99)	14.7
	RV GLS %	0.26 (2.14)	0.97 (0.94–0.99)	7.9

SD: standard deviation. ICC: intraclass correlation coefficient. CoV: coefficient of variation. LV: left ventricular. RV: right ventricular. GLS: global longitudinal strain. GCS: global circumferential strain. GRS: global radial strain.

**Fig 2:**
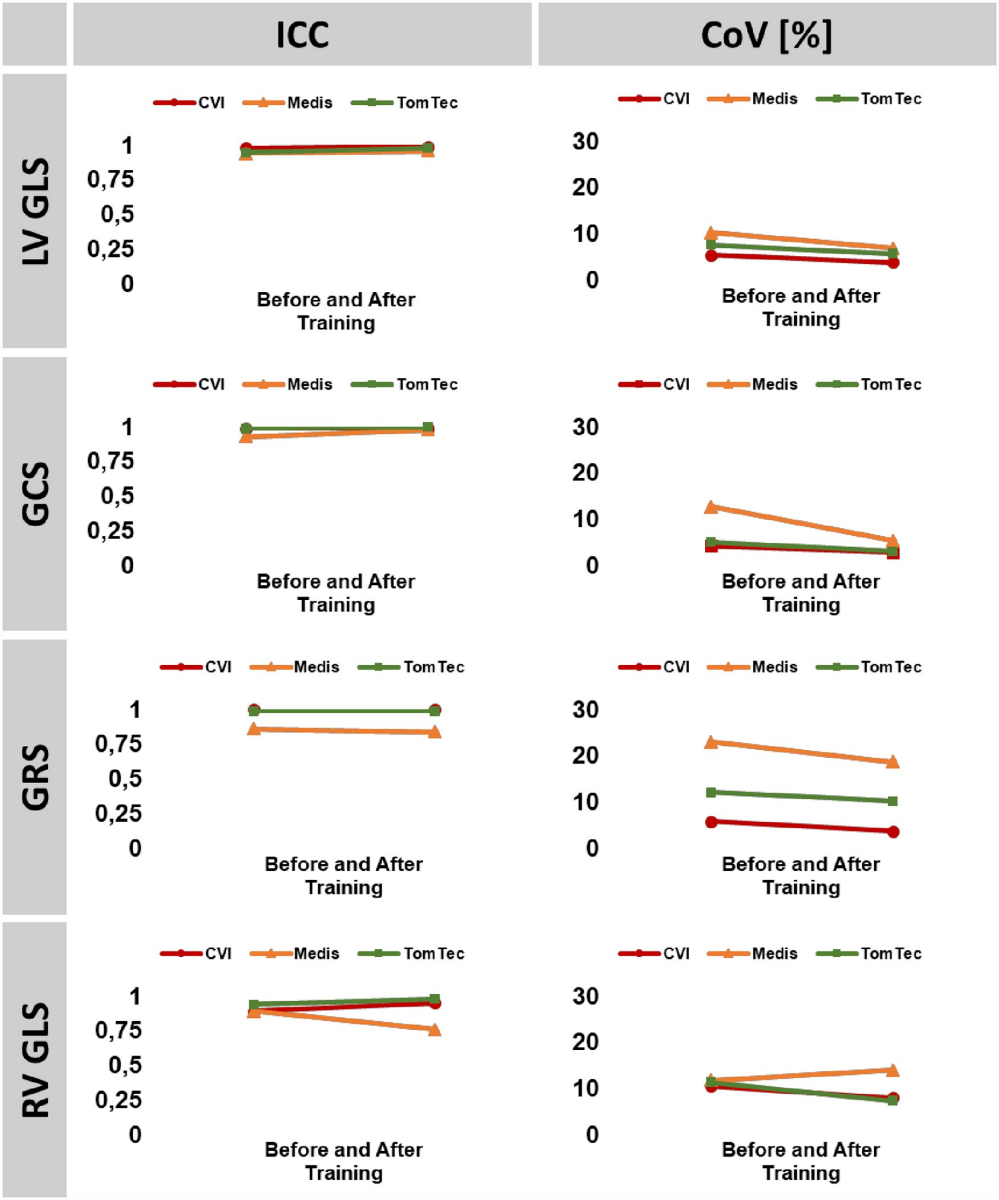
Intra-observer reproducibility. The graph shows interclass correlation coefficients (ICC) and coefficients of variation (CoV) for intra-observer reproducibility prior to and after training. LV/RV: left/right ventricle, GLS: global longitudinal strain, GCS: global circumferential strain, GRS: global radial strain.

**Fig 3:**
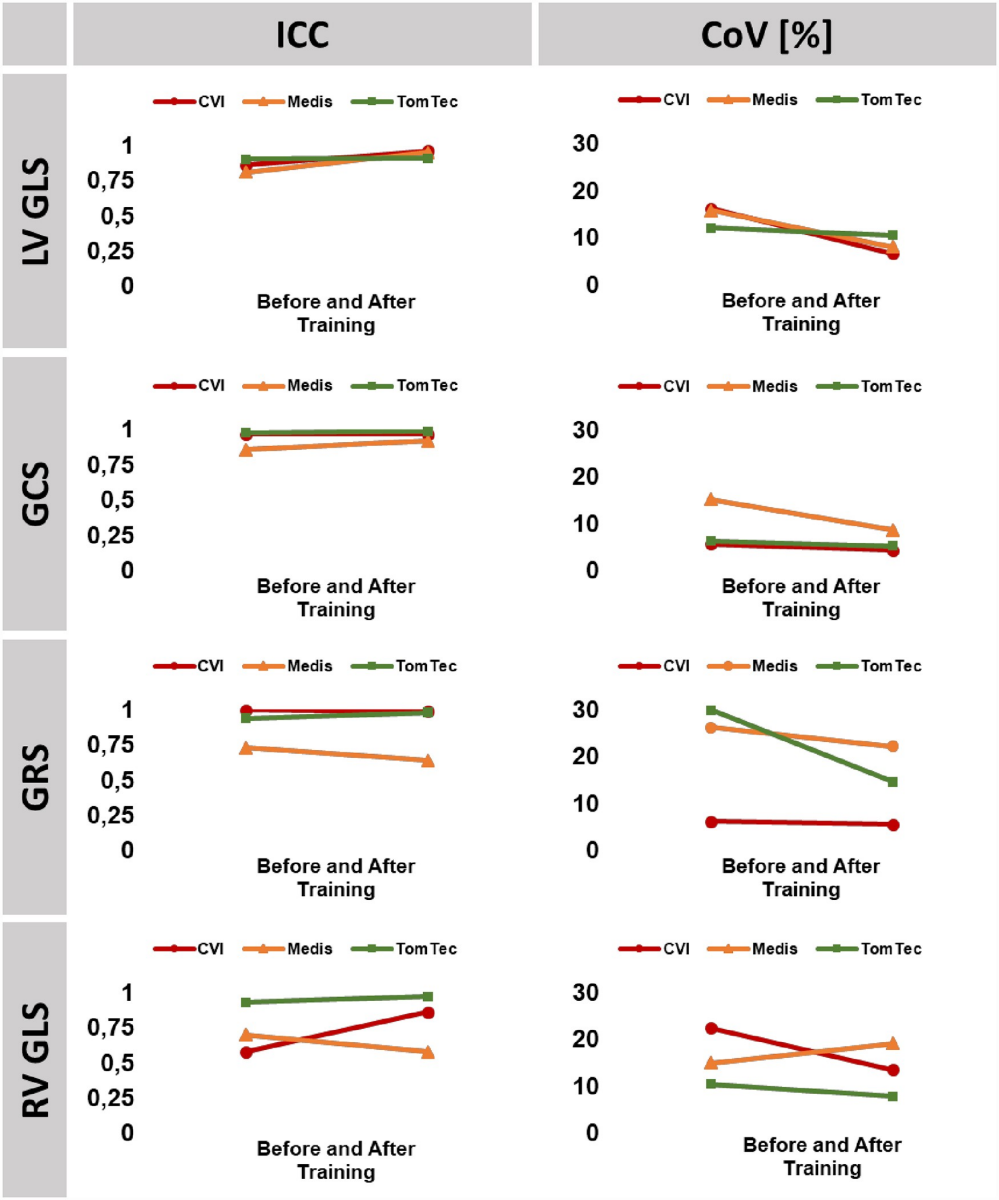
Inter-observer reproducibility. The graph shows interclass correlation coefficients (ICC) and coefficients of variation (CoV) for inter-observer reproducibility prior to and after training. LV/RV: left/right ventricle, GLS: global longitudinal strain, GCS: global circumferential strain, GRS: global radial strain.

GCS and LV GLS were the most robust parameters prior to training with sufficient overall reproducibility, highest in CVI followed by TomTec and Medis. Training further improved reproducibility with higher impact on inter-observer reproducibility, achieving similar results within each software solution employed (Tables 2 to 4). Within LV strain parameters, GRS was the least robust with lower reproducibility depending on different software solutions employed. RV GLS reproducibility was low. Whilst training significantly improved RV GLS reproducibility based on CVI and TomTec analyses it had no positive effect on Medis based reproducibility of RV GLS (Figs 2 and 3).

### Health and disease

Prior to training, there was a distinct pattern of better LV strain reproducibility in patients compared to healthy volunteers. This difference was less apparent after training with subsequent improved reproducibility within patients and volunteers (S2 to S4 Tables). RV GLS showed no clear difference in variance between patients and healthy volunteers.

### Inter-vendor agreement

**Fig 4:**
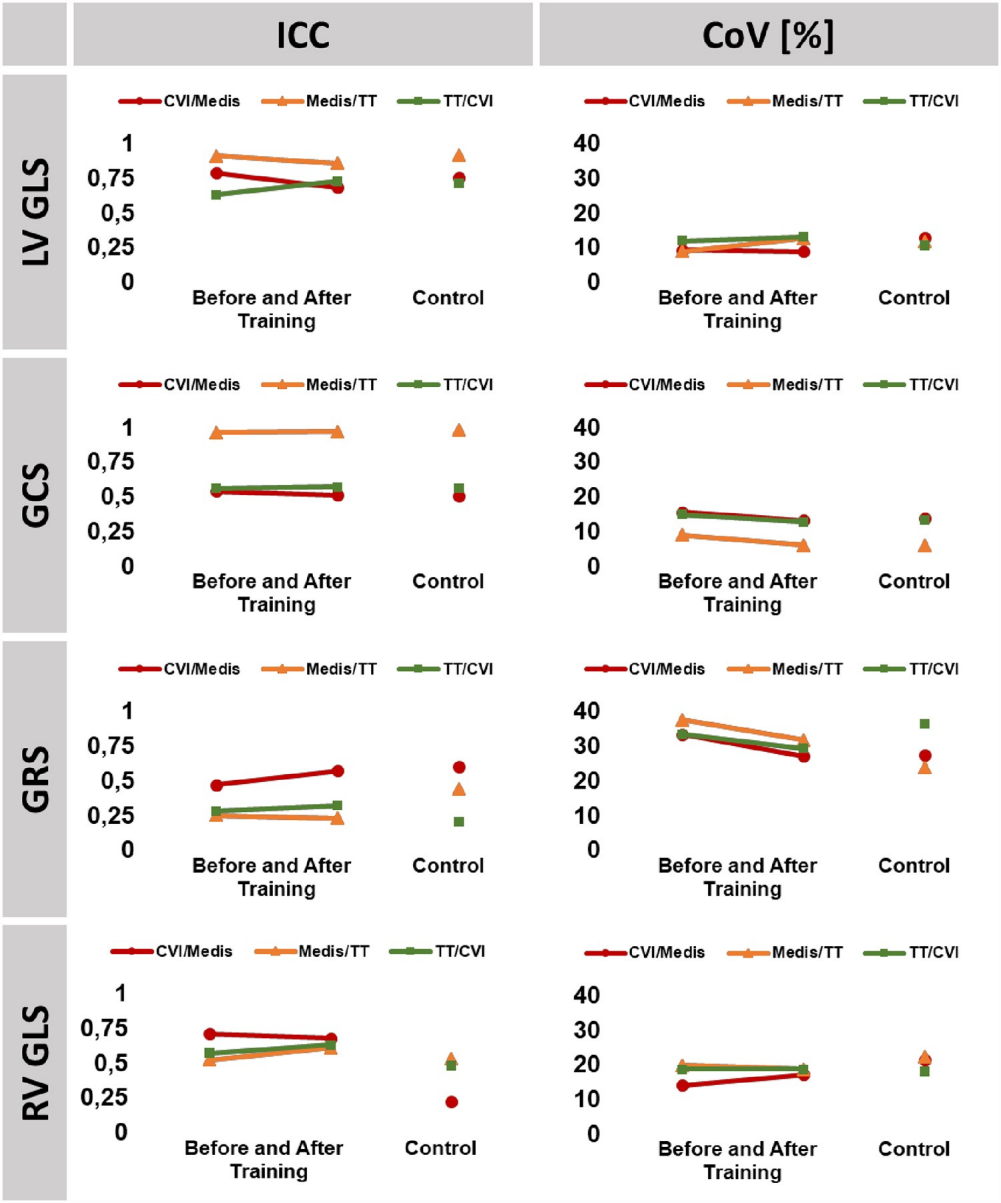
Inter-vendor reproducibility. The graph shows interclass correlation coefficients (ICC) and coefficients of variation (CoV) for inter-vendor agreement prior to and after training as well as the agreement of a trained investigator (control) as a reference. LV/RV: left/right ventricle, GLS: global longitudinal strain, GCS: global circumferential strain, GRS: global radial strain.

Agreement was excellent for GCS closely followed by LV GLS between TomTec and Medis and good (LV GLS) to fair (GCS) comparing either of them to CVI. Training did not distinctly increase absolute inter-vendor agreements. In contrast, inter-vendor agreement was globally weak to fair comparing GRS and RV GLS. Reproducibility after training was similar to reproducibility of an experienced investigator at the core laboratory (Fig 4).

## Discussion

The present study reports the impact of training on the reproducibility of CMR-FT derived myocardial deformation assessment and bears several important findings. First, training increases operator reproducibility independently of FT software and cardiac function. Second, LV GLS and GCS were the most robust parameters with excellent reproducibility already before dedicated training. Third, impact of training was higher for RV than LV assessments. Forth, training resulted in a statistically significant change in some strain parameters obtained with some of the software solutions. Last, dedicated training increases strain reproducibility to a level that is comparable to experienced CMR-FT operators and considering the easy and fast computation based on routinely acquired b-SSFP sequences, CMR-FT may consequently be fully implemented into clinical routine MRI evaluations. However, since there is significant variance introduced by different software solutions, currently these novel parameters should be quantified using a given software if serial assessments or follow-up acquisitions are required.

### Observer experience—Impact of training & reliability of feature tracking

Clinical studies report GLS to be of high potential for clinical decision making and value for mortality prediction beyond established risk factors such as LVEF [10–12, 14, 18]. Since CMR-FT provides easy, fast and accurate evaluation of cardiac myocardial deformation [5, 7], myocardial strain seems useful for broad clinical application. However, these data stem from highly trained core laboratories. The current study now reports real-world reproducibility data of the existing deformation parameters for the three most utilized software solutions and defines the value of appropriate observer training. In fact, volumetric approaches such as LVEF are commonly established for cardiovascular risk assessment [26–28] and impact of training has also been previously reported [22]. The impact of training on LV GLS and GCS can be appreciated from subsequent improved reproducibility amongst all software solutions tested. Despite being the most robust parameters prior to and after training, the latter not only improved reproducibility but also resulted in a statistically significant change in absolute LV GLS and GCS values (Table 1), both of which are critically considered for clinical routine implementation due to increasing prognostic value [11, 14]. However, changes in absolute strain values amount to a maximum of 1.5%. Further studies are warranted to assess the clinical relevance of this finding.

Measurement bias is largely attributed to intra- and inter-observer variability [20–22], consequently training plays a pivotal role when standardized analyses are required. Feisst et al. [23] showed the direct influence of observer experience (0 vs. 1.5 vs. 5 years of experience) on FT reproducibility. Within their study intra-observer reproducibility of GCS was highest in the most experienced observer showing similar reproducibility to that achieved by operators after dedicated training using any of the three software types in the current study.

Interestingly, GCS closely followed by LV GLS showed software independent excellent reproducibility before training. This adds to the current literature available on reproducibility in CMR-FT [7, 9, 24], demonstrating both LV GLS and GCS as the most robust strain parameters. Furthermore, averaging strain values from 3 repetitions has proven usefulness regarding reproducibility and reliability [8, 9] and may partly explain the sufficient reproducibility in untrained operators in the current study.

### Cardiac anatomy and function

Studies providing evidence for the clinical benefits of RV GLS assessment in arrhythmogenic right ventricular cardiomyopathy [29] or pulmonary hypertension [30] emphasize the need for reliable RV strain assessments. Worse reproducibility for RV [7, 24] compared to LV assessment and beneficial effects of training have been demonstrated for volumetry [22]. The thin walled RV with lower myocardial mass and higher degree of trabeculation as well as the position of the pulmonary valve are discussed as potential causes of higher variability. Additionally, the 4 CV is more affected by breathing motion. Hence, RV assessment solely in the 4 CV is more susceptible to insufficient tracking especially by inexperienced operators, limiting clinical applications. The current data provides evidence of training impact leading to significantly improved RV analyses, which can be appreciated by marked reduction in RV GLS variance to a level comparable to LV parameters using TomTec and CVI. Medis on the other hand showed worse reproducibility, which is in apparent contradiction to previous data showing better reproducibility in the RV as compared to TomTec [9]. It is interesting to speculate whether the greater amount of user interaction possible with Medis with potential manipulation of end-diastolic and end-systolic contours leads to better reproducibility in experienced hands [9] but conversely represents a source of increased variance when used in inexperienced hands. Notwithstanding, the complex nature of RV anatomy represents a challenge for any strain assessment and it is promising to see that reproducibility can be improved by training [7, 9, 24].

Possibly as a result of less complex LV anatomy LV function is easier to assess using deformation imaging. Because of better reproducibility LV strain has been adopted more in clinical routine and recent literature suggests superior value of LV myocardial strain in mortality prediction as compared to LVEF [11, 12, 14, 16]. Noteworthy, intra- and inter-observer reproducibility of LV strains is higher in HF patients compared to healthy volunteers prior to training, which has been previously reported for speckle-tracking echocardiography [31] and CMR deformation analyses [32]. Higher strain values as well as LVEF of healthy subjects are associated with higher cardiac motion, which may negatively impact reproducibility and may require more operator experience. One of the underlying reasons is increased through plane motion [3, 7, 33] with more features leaving the 2D imaging plane during the cardiac cycle. However, the difference of reproducibility between healthy volunteers and patients attenuated after training resulting in consistently improved reproducibility for LV strains, suggesting that training and standard operator assessment has an important effect in more challenging test conditions. There was no distinct pattern for better RV reproducibility in health or disease, which is likely explained by its higher inherent variability [7, 9].

### Technical considerations

Whilst visual interpretation of wall motion in CMR cine sequences is likely inter-vendor independent, it is highly susceptible to differences in observer experience [20]. Feature tracking is based on optical flow technology [34] identifying different anatomical elements at the cavity-myocardial boundary and subsequently following them over a cardiac cycle by a method of maximum likelihood [4]. Although the introduction of quantitative myocardial deformation assessment provided an less observer dependant approach as demonstrated during dobutamine stress [35], individual vendor specific differences in the underlying algorithms are undisclosed [3]. CVI was the most reliable software for untrained operators in the current study. Corrections to the initial contour were rarely needed, resulting in high initial reproducibility for untrained operators. TomTec showed better initial reproducibility for LV and RV strain values compared to Medis, which again could be due to the necessity of more user-interaction using Medis. Nevertheless, it is important to note that dedicated observer training significantly increases reproducibility and may have greater benefit when more user-interaction is required. As a result one may argue that an automated analysis with as little operator dependency as possible may be desirable. The level of variance between software types however was not significantly improved by training with similar results for newly trained and experienced operators [9] suggesting software introduced variance rather than observer-dependence [36]. This may have clinical impact as demonstrated by Eitel et al. [14] who showed superior prognostic value of GLS (based on TomTec) over LVEF in patients after myocardial infarction as opposed to Gavara et al. [18] who failed to reproduce these results based on CVI.

Due to significant variations in strain values obtained by different software solutions and measurement techniques, to date these novel parameters should be quantified similarly if serial assessments or follow-up acquisitions are required. Similar problems have been successfully addressed in echocardiographical speckle-tracking with efforts being directed towards consensus [37] trying to standardize technology to enable wider interchangeability and comparisons. Therefore, more clinical trials are needed, evaluating the technical and software specific properties in CMR imaging, to reliably introduce standardisation and reference values for myocardial deformation assessment for widespread clinical introduction. In this context, dedicated observer training will play an essential role to reliably address some of the associated challenges.

## Study limitations

The results are based on CMR-FT data with no echocardiographic or CMR-tagging reference standard. However, the conclusions are derived from the comparison of the three most commonly used software solutions for detailed myocardial deformation assessments. The study collective consisted of 12 HF patients and 12 healthy volunteers for statistical evaluation, which may not be necessarily large enough for sub-group comparison for health and disease. Nevertheless, we are able to demonstrate beneficial impact of training both in patients and healthy volunteers. Although no patients were studied at 3T, similar findings may be expected at 3T considering previously described similar reproducibility of CMR-FT at 1.5 and 3T [38]. Furthermore, notwithstanding previous reports showing similar reproducibility for FT in DCM, HCM and LV hypertrophy, we cannot exclude that training effects may differ in impact in these populations.

## Conclusion

Training increases reproducibility of CMR-FT derived strain parameters independent of software solution or cardiac function. LV GLS and GCS are the most robust parameters with highest independence of observer experience. Efforts need to be directed towards technical and clinical standardisation to allow for implementation of reference values irrespective of utilized software solutions. In this context, dedicated observer training will play a pivotal role to further reduce observer dependence and allow for broad adoption of this technology into routine clinical use.

## Supporting information

S1 Table Strain in healthy volunteers and heart failure patients. Continuous variables are expressed as mean (standard deviation). The Wilcoxon signed-rank test was used to determine significant differences for continuous and the chi-squared test for categorial variables. LVEF/RVEF, left/right ventricular ejection fraction; GLS/GCS/GRS, global longitudinal/circumferential/radial strain.
(DOCX)

S2 Table Intra- and inter-observer reproducibility using CVI prior to and after training for healthy volunteers and patients. SD: standard deviation. ICC: intraclass correlation coefficient. CoV: coefficient of variation. LV: left ventricular. RV: right ventricular. GLS: global longitudinal strain. GCS: global circumferential strain. GRS: global radial strain.
(DOCX)

S3 Table Intra- and inter-observer reproducibility using medis prior to and after training for healthy volunteers and patients. SD: standard deviation. ICC: intraclass correlation coefficient. CoV: coefficient of variation. LV: left ventricular. RV: right ventricular. GLS: global longitudinal strain. GCS: global circumferential strain. GRS: global radial strain.
(DOCX)

S4 Table Intra- and inter-observer reproducibility using TomTec prior to and after training for healthy volunteers and patients. SD: standard deviation. ICC: intraclass correlation coefficient. CoV: coefficient of variation. LV: left ventricular. RV: right ventricular. GLS: global longitudinal strain. GCS: global circumferential strain. GRS: global radial strain.
(DOCX)

S1 Fig Intra-observer reproducibility prior and after teaching with CVI. Bland Altman plots are shown for the study collective prior to and after training using CVI. LV/RV: left/right ventricle, GLS: global longitudinal strain, GCS: global circumferential strain, GRS: global radial strain, Δ: difference.
(DOCX)

S2 Fig Inter-observer reproducibility prior and after teaching with CVI. Bland Altman plots are shown for the study collective prior to and after training using CVI. LV/RV: left/right ventricle, GLS: global longitudinal strain, GCS: global circumferential strain, GRS: global radial strain, Δ: difference.
(DOCX)

S3 Fig Intra-observer reproducibility prior and after teaching with Medis. Bland Altman plots are shown for the study collective prior to and after training using Medis. LV/RV: left/right ventricle, GLS: global longitudinal strain, GCS: global circumferential strain, GRS: global radial strain, Δ: difference.
(DOCX)

S4 Fig Inter-observer reproducibility prior and after teaching with Medis. Bland Altman plots are shown for the study collective prior to and after training using Medis. LV/RV: left/right ventricle, GLS: global longitudinal strain, GCS: global circumferential strain, GRS: global radial strain, Δ: difference.
(DOCX)

S5 Fig Intra-observer reproducibility prior and after teaching with TomTec. Bland Altman plots are shown for the study collective prior to and after training using TomTec. LV/RV: left/right ventricle, GLS: global longitudinal strain, GCS: global circumferential strain, GRS: global radial strain, Δ: difference.
(DOCX)

S6 Fig Inter-observer reproducibility prior and after teaching with TomTec. Bland Altman plots are shown for the study collective prior to and after training using TomTec. LV/RV: left/right ventricle, GLS: global longitudinal strain, GCS: global circumferential strain, GRS: global radial strain, Δ: difference.
(DOCX)

## List of abbreviations

CMR: cardiovascular magnetic resonance
CMR-FT: CMR feature tracking
CoV: coefficient of variation
HFpEF: heart failure with preserved ejection fraction
ICC: intra-class correlation coefficient
LV: left ventricle
LVEF: left ventricular ejection fraction
MD: mean difference
RV: right ventricle
SA: short axis
SD: standard deviation
