# Supplementary material for: Cardiovascular magnetic resonance imaging feature tracking: Impact of training on observer performance and reproducibility
Source: PLoS One. 2019 Jan 25;14(1):e0210127. doi: 10.1371/journal.pone.0210127 (PMC6347155; doi:10.1371/journal.pone.0210127)
Supplement: S1 Table — Continuous variables are expressed as mean (standard deviation). The Wilcoxon signed-rank test was used to determine significant differences for continuous and the chi-squared test for categorial variables. LVEF/RVEF, left/right ventricular ejection fraction; GLS/GCS/GRS, global longitudinal/circumferential/radial strain. (DOCX) [file pone.0210127.s001.docx]

| Study population (n) | | Healthy volunteers (12) | |  | HFpEF patients (12) | |  |
| --- | --- | --- | --- | --- | --- | --- | --- |
| Gender (F/M) |  | 6/6 | |  | 5/7 | | 0.682 |
| Age (years) |  | 24 (5.0) | |  | 74 (6.5) | | <0.001 |
| LVEF (%) |  | 60 (1.28) | |  | 50 (12.72) | | 0.039 |
|  | Software | Before Training | After Training | p | Before Training | After Training | p |
| LV GLS (%) | CVI | -17.46 (2.89) | -17.87 (1.11) | 0.879 | -14.01 (2.80) | -14.46 (2.83) | **0.041** |
|  | Medis | -20.11 (1.57) | -21.57 (1.90) | **<0.001** | -16.71 (4.26) | -18.18 (4.07) | **0.002** |
|  | TomTec | -21.99 (2.68) | -20.27 (2.61) | **0.018** | -18.16 (4.43) | -17.98 (4.06) | 0.820 |
| GCS (%) | CVI | -20.20 (1.66) | -20.26 (1.65) | 0.668 | -16.62 (4.22) | -16.63 (4.75) | 0.637 |
|  | Medis | -30.04 (3.23) | -30.63 (3.15) | 0.530 | -25.88 (9.11) | -26.24 (8.48) | 0.290 |
|  | TomTec | -27.98 (2.71) | -28.48 (2.82) | **0.017** | -22.72 (7.03) | -22.97 (6.90) | 0.449 |
| GRS (%) | CVI | 39.25 (5.09) | 39.21 (4.90) | 0.954 | 32.47 (10.51) | 33.75 (11.51) | **0.01** |
|  | Medis | 56.22 (13.95) | 59.23 (8.53) | 0.290 | 44.37 (14.61) | 42.15 (16.68) | 0.209 |
|  | TomTec | 27.43 (8.66) | 24.65 (6.12) | 0.092 | 23.23 (9.16) | 23.25 (6.66) | 0.549 |
| RV GLS (%) | CVI | -23.69 (4.90) | -24.23 (3.62) | 0.648 | -22.11 (3.69) | -21.74 (3.58) | 0.587 |
|  | Medis | -24.13 (3.59) | -25.45 (3.66) | 0.072 | -23.06 (5.42) | -23.20 (6.17) | 0.584 |
|  | TomTec | -28.59 (3.87) | -28.72 (5.03) | 0.954 | -24.56 (5.27) | -25.07 (5.11) | 0.376 |

**S1 Table. Strain in healthy volunteers and heart failure patients.**

Continuous variables are expressed as mean (standard deviation). The Wilcoxon signed-rank test was used to determine significant differences for continuous and the chi-squared test for categorial variables. LVEF/RVEF, left/right ventricular ejection fraction; GLS/GCS/GRS, global longitudinal/circumferential/radial strain.
