# Supplementary material for: Cardiovascular magnetic resonance imaging feature tracking: Impact of training on observer performance and reproducibility
Source: PLoS One. 2019 Jan 25;14(1):e0210127. doi: 10.1371/journal.pone.0210127 (PMC6347155; doi:10.1371/journal.pone.0210127)
Supplement: S2 Table — SD: standard deviation. ICC: intraclass correlation coefficient. CoV: coefficient of variation. LV: left ventricular. RV: right ventricular. GLS: global longitudinal strain. GCS: global circumferential strain. GRS: global radial strain. (DOCX) [file pone.0210127.s002.docx]

| Software: CVI | Strain | Volunteers Mean Difference (SD of the Diff.) | ICC (95% CI) | CoV (%) | Patients Mean Difference (SD of the Diff.) | ICC (95% CI) | CoV (%) |
| --- | --- | --- | --- | --- | --- | --- | --- |
| Intra-observer | LV GLS % | 0.18 (0.86) | 0.79 (0.28-0.94) | 4.9 | -0.12 (0.88) | 0.98 (0.91-0.99) | 6.2 |
| before Training | GCS % | -0.36 (0.89) | 0.93 (0.76-0.98) | 4.5 | 0.21 (0.44) | 1 (0.99-1) | 2.7 |
|  | GRS % | 1.59 (2.09) | 0.96 (0.87-0.99) | 5.5 | -0.67 (1.20) | 1 (0.99-1) | 3.7 |
|  | RV GLS % | -0.26 (3.10) | 0.80 (0.31-0.94) | 12.6 | 0.72 (1.70) | 0.87 (0.53-0.96) | 7.4 |
|  |  |  |  |  |  |  |  |
| Intra-observer | LV GLS % | 0.38 (0.65) | 0.88 (0.59-0.97) | 3.7 | -0.02 (0.49) | 0.99 (0.98-1) | 3.4 |
| after Training | GCS % | 0.00 (0.28) | 0.99 (0.97-1) | 1.4 | 0.48 (0.62) | 1 (0.99-1) | 3.8 |
|  | GRS % | -0.05 (1.23) | 0.98 (0.94-1) | 3.3 | -0.68 (1.35) | 1 (0.99-1) | 4.1 |
|  | RV GLS % | -0.73 (2.01) | 0.94 (0.80-0.98) | 8.4 | 1.08 (1.20) | 0.97 (0.90-0.99) | 5.4 |
|  |  |  |  |  |  |  |  |
| Inter-observer | LV GLS % | -0.31 (3.56) | 0.43 (0.0-0.84) | 20.4 | -0.50 (0.98) | 0.97 (0.89-0.99) | 7.0 |
| before Training | GCS % | 0.35 (0.94) | 0.92 (0.71-0.98) | 4.6 | 0.78 (1.15) | 0.98 (0.94-1) | 6.9 |
|  | GRS % | -0.79 (2.68) | 0.93 (0.75-0.98) | 6.8 | -0.90 (1.85) | 0.99 (0.97-1) | 5.7 |
|  | RV GLS % | -2.26 (5.97) | 0.4 (0.0-0.83) | 25.2 | -1.10 (4.34) | 0.49 (0.0-0.85) | 19.6 |
|  |  |  |  |  |  |  |  |
| Inter-observer | LV GLS % | 0.90 (0.81) | 0.82 (0.36-0.95) | 4.5 | 0.30 (1.22) | 0.95 (0.84-0.99) | 8.5 |
| after Training | GCS % | 1.02 (0.37) | 0.99 (0.95-1) | 1.8 | 1.14 (1.12) | 0.99 (0.95-1) | 6.8 |
|  | GRS % | -3.63 (2.12) | 0.95 (0.81-0.98) | 5.4 | -2.73 (1.99) | 0.99 (0.98-1) | 5.9 |
|  | RV GLS % | -0.03 (4.25) | 0.51 (0.0-0.86) | 17.5 | -0.40 (1.50) | 0.96 (0.85-0.99) | 6.9 |

**S2 Table. Intra- and Inter-observer reproducibility using CVI prior to and after training** **for healthy volunteers and patients.**

SD: standard deviation. ICC: intraclass correlation coefficient. CoV: coefficient of variation. LV: left ventricular. RV: right ventricular. GLS: global longitudinal strain. GCS: global circumferential strain. GRS: global radial strain.
