# Supplementary material for: Cardiovascular magnetic resonance imaging feature tracking: Impact of training on observer performance and reproducibility
Source: PLoS One. 2019 Jan 25;14(1):e0210127. doi: 10.1371/journal.pone.0210127 (PMC6347155; doi:10.1371/journal.pone.0210127)
Supplement: S3 Table — SD: standard deviation. ICC: intraclass correlation coefficient. CoV: coefficient of variation. LV: left ventricular. RV: right ventricular. GLS: global longitudinal strain. GCS: global circumferential strain. GRS: global radial strain. (DOCX) [file pone.0210127.s003.docx]

| Software: Medis | Strain | Volunteers Mean Difference (SD of the Diff.) | ICC (95% CI) | CoV (%) | Patients Mean Difference (SD of the Diff.) | ICC (95% CI) | CoV (%) |
| --- | --- | --- | --- | --- | --- | --- | --- |
| Intra-observer | LV GLS % | -0.03 (1.77) | 0.53 (0.0-0.86) | 8.4 | -0.05 (2.12) | 0.94 (0.78-0.98) | 12.8 |
| before Training | GCS % | -2.88 (3.37) | 0.37 (0.0-0.82) | 12.0 | 0.95 (1.88) | 0.99 (0.95-1) | 7.9 |
|  | GRS % | -3.80 (12.9) | 0.69 (0-0.91) | 23.1 | 2.17 (7.93) | 0.9 (0.66-0.97) | 20.2 |
|  | RV GLS % | 0.13 (2.35) | 0.87 (0.54-0.96) | 10.0 | 0.62 (2.90) | 0.88 (0.59-0.97) | 13.8 |
|  |  |  |  |  |  |  |  |
| Intra-observer | LV GLS % | -0.27 (1.66) | 0.42 (0.0-0.83) | 7.8 | 0.09 (1.01) | 0.99 (0.95-1) | 5.5 |
| after Training | GCS % | -1.98 (1.03) | 0.95 (0.83-0.99) | 3.6 | -0.45 (1.38) | 0.99 (0.97-1) | 5.8 |
|  | GRS % | 7.07 (5.73) | 0.53 (0-0.87) | 11.4 | 2.85 (6.80) | 0.82 (0.37-0.95) | 19.4 |
|  | RV GLS % | -0.06 (1.80) | 0.89 (0.62-0.97) | 7.4 | -0.08 (4.29) | 0.59 (0.0-0.88) | 19.7 |
|  |  |  |  |  |  |  |  |
| Inter-observer | LV GLS % | -1.73 (1.70) | 0.31 (0.0-0.8) | 8.5 | 0.28 (3.36) | 0.8 (0.30-0.94) | 21.3 |
| before Training | GCS % | 1.03 (3.95) | 0.42 (0.0-0.83) | 13.1 | 4.86 (3.80) | 0.95 (0.84-0.97) | 14.7 |
|  | GRS % | -4.73 (15.05) | 0.60 (0-0.88) | 26.8 | -8.24 (11.57) | 0.80 (0.32-0.94) | 26.1 |
|  | RV GLS % | 1.53 (2.16) | 0.90 (0.65-0.97) | 9.0 | 4.59 (4.06) | 0.80 (0.32-0.94) | 17.6 |
|  |  |  |  |  |  |  |  |
| Inter-observer | LV GLS % | -0.01 (2.05) | 0.61 (0.0-0.89) | 9.5 | 0.08 (1.10) | 0.98 (0.94-1) | 6.0 |
| after Training | GCS % | 1.75 (1.67) | 0.92 (0.72-0.98) | 5.5 | 3.95 (2.72) | 0.97 (0.91-0.99) | 10.4 |
|  | GRS % | -10.97 (6.04) | 0.73 (0.08-0.92) | 10.2 | -16.96 (14.44) | 0.68 (0.0-0.91) | 34.3 |
|  | RV GLS % | 1.81 (4.81) | 0.52 (0.0-0.86) | 19.0 | 4.85 (4.30) | 0.80 (0.29-0.94) | 17.8 |

**S3 Table. Intra- and Inter-observer reproducibility using Medis prior to and after training** **for healthy volunteers and patients.**

SD: standard deviation. ICC: intraclass correlation coefficient. CoV: coefficient of variation. LV: left ventricular. RV: right ventricular. GLS: global longitudinal strain. GCS: global circumferential strain. GRS: global radial strain.
