# Supplementary material for: Cardiovascular magnetic resonance imaging feature tracking: Impact of training on observer performance and reproducibility
Source: PLoS One. 2019 Jan 25;14(1):e0210127. doi: 10.1371/journal.pone.0210127 (PMC6347155; doi:10.1371/journal.pone.0210127)
Supplement: S4 Table — SD: standard deviation. ICC: intraclass correlation coefficient. CoV: coefficient of variation. LV: left ventricular. RV: right ventricular. GLS: global longitudinal strain. GCS: global circumferential strain. GRS: global radial strain. (DOCX) [file pone.0210127.s004.docx]

| Software: TomTec | Strain | Volunteers Mean Difference (SD of the Diff.) | ICC (95% CI) | CoV (%) | Patients Mean Difference (SD of the Diff.) | ICC (95% CI) | CoV (%) |
| --- | --- | --- | --- | --- | --- | --- | --- |
| Intra-observer | LV GLS % | -1.98 (1.74) | 0.86 (0.50-0.96) | 8.0 | -0.49 (0.76) | 0.99 (0.98-1) | 4.2 |
| before Training | GCS % | -0.18 (0.81) | 0.98 (0.92-0.99) | 2.9 | -0.88 (1.62) | 0.99 (0.95-1) | 7.1 |
|  | GRS % | 0.14 (3.44) | 0.91 (0.69-0.97) | 13.5 | -1.55 (2.12) | 0.98 (0.94-1) | 9.3 |
|  | RV GLS % | 0.4 (2.91) | 0.90 (0.66-0.972) | 10.4 | -0.46 (3.03) | 0.91 (0.69-0.98) | 12.7 |
|  |  |  |  |  |  |  |  |
| Intra-observer | LV GLS % | 0.0 (1.26) | 0.99 (0.98-1) | 6.5 | 0.0 (0.90) | 0.99 (0.96-1) | 5.0 |
| after Training | GCS % | 0.28 (0.72) | 0.98 (0.94-1) | 2.5 | 0.42 (0.91) | 1 (0.99-1) | 4.0 |
|  | GRS % | 0.94 (2.88) | 0.98 (0.92-0.99) | 11.2 | 1.90 (1.97) | 0.98 (0.92-0.99) | 8.7 |
|  | RV GLS % | 0.33 (1.47) | 0.98 (0.93-0.99) | 5.0 | -0.17 (2.38) | 0.96 (0.85-0.99) | 9.7 |
|  |  |  |  |  |  |  |  |
| Inter-observer | LV GLS % | -1.90 (2.35) | 0.73 (0.07-0.92) | 10.7 | -0.10 (2.26) | 0.93 (0.77-0.98) | 12.5 |
| before Training | GCS % | -0.02 (0.82) | 0.98 (0.92-0.99) | 2.9 | -1.21 (1.98) | 0.98 (0.93-0.99) | 8.7 |
|  | GRS % | -3.62 (8.14) | 0.72 (0.01-0.92) | 29.7 | -2.81 (7.35) | 0.81 (0.35-0.95) | 31.6 |
|  | RV GLS % | 1.58 (2.13) | 0.92 (0.72-0.98) | 7.4 | 1.12 (3.39) | 0.89 (0.61-0.97) | 13.8 |
|  |  |  |  |  |  |  |  |
| Inter-observer | LV GLS % | 2.07 (2.00) | 0.8 (0.30-0.94) | 9.8 | -0.02 (1.42) | 0.97 (0.9-0.99) | 7.9 |
| after Training | GCS % | 0.10 (1.13) | 0.96 (0.86-0.99) | 4.0 | 0.71 (1.56) | 0.99 (0.96-1) | 6.8 |
|  | GRS % | 3.16 (3.43) | 0.91 (0.70-0.98) | 13.9 | 0.58 (3.27) | 0.94 (0.79-0.98) | 14.1 |
|  | RV GLS % | -0.56 (1.80) | 0.97 (0.89-0.99) | 6.3 | 1.07 (2.20) | 0.95 (0.84-0.99) | 8.8 |

**S4 Table. Intra- and Inter-observer reproducibility using TomTec prior to and after training for healthy volunteers and patients.**

SD: standard deviation. ICC: intraclass correlation coefficient. CoV: coefficient of variation. LV: left ventricular. RV: right ventricular. GLS: global longitudinal strain. GCS: global circumferential strain. GRS: global radial strain.
