# Supplementary material for: Cardiovascular magnetic resonance imaging feature tracking: Impact of training on observer performance and reproducibility
Source: PLoS One. 2019 Jan 25;14(1):e0210127. doi: 10.1371/journal.pone.0210127 (PMC6347155; doi:10.1371/journal.pone.0210127)
Supplement: S1 Fig — Bland Altman plots are shown for the study collective prior to and after training using CVI. LV/RV: left/right ventricle, GLS: global longitudinal strain, GCS: global circumferential strain, GRS: global radial strain, Δ: difference. (DOCX) [file pone.0210127.s005.docx]

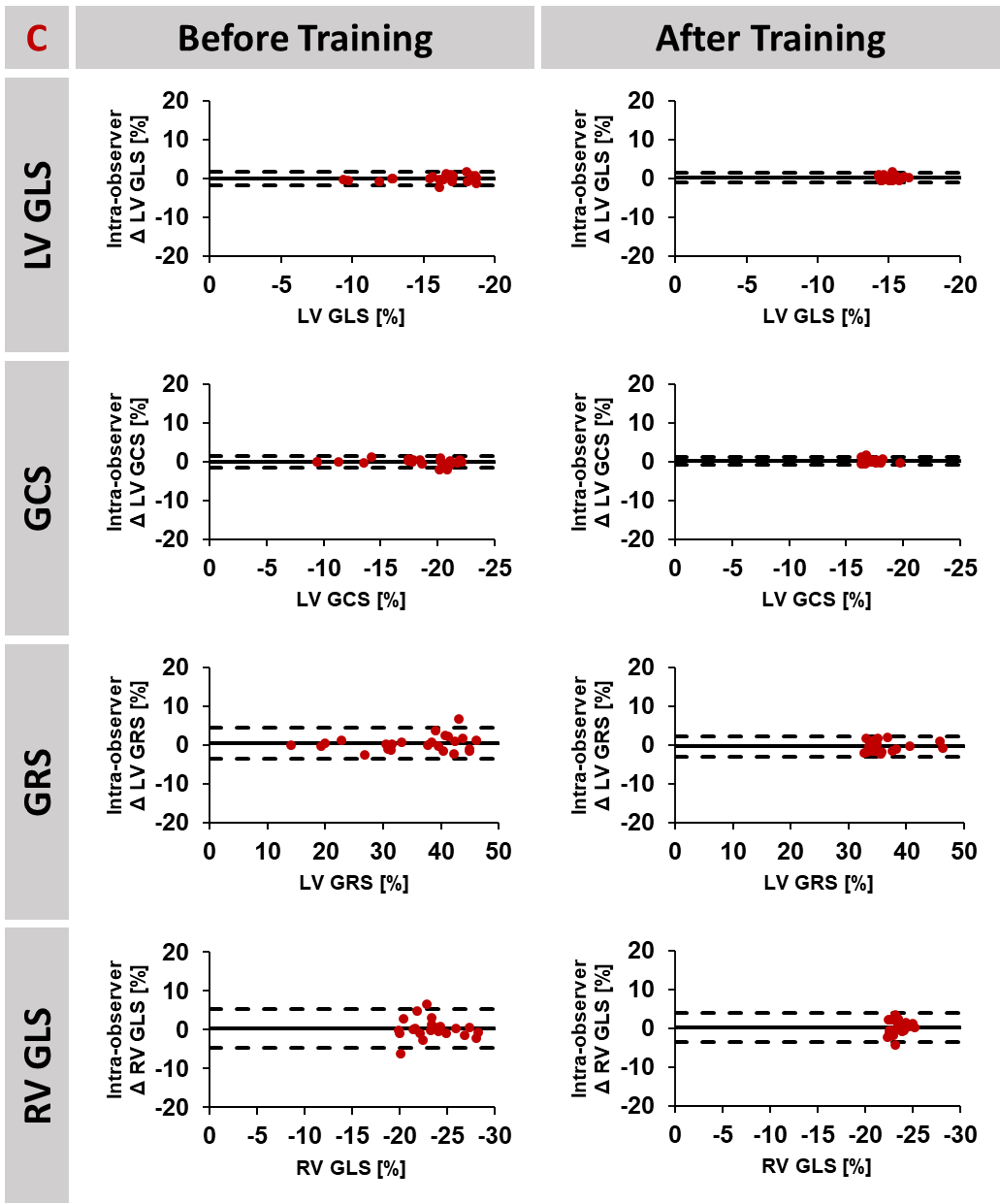


**S1 Fig Intra-observer reproducibility prior and after teaching with CVI**

Bland Altman plots are shown for the study collective prior to and after training using CVI. LV/RV: left/right ventricle, GLS: global longitudinal strain, GCS: global circumferential strain, GRS: global radial strain, Δ: difference.
